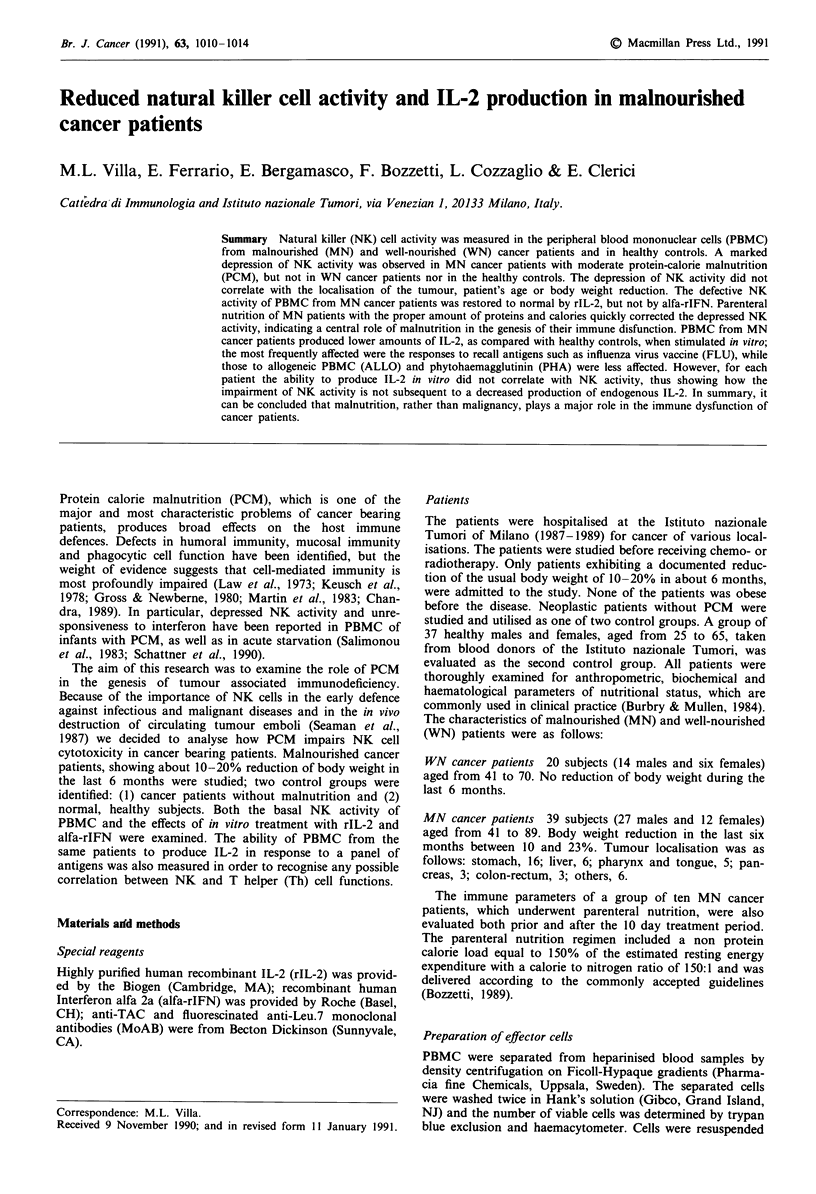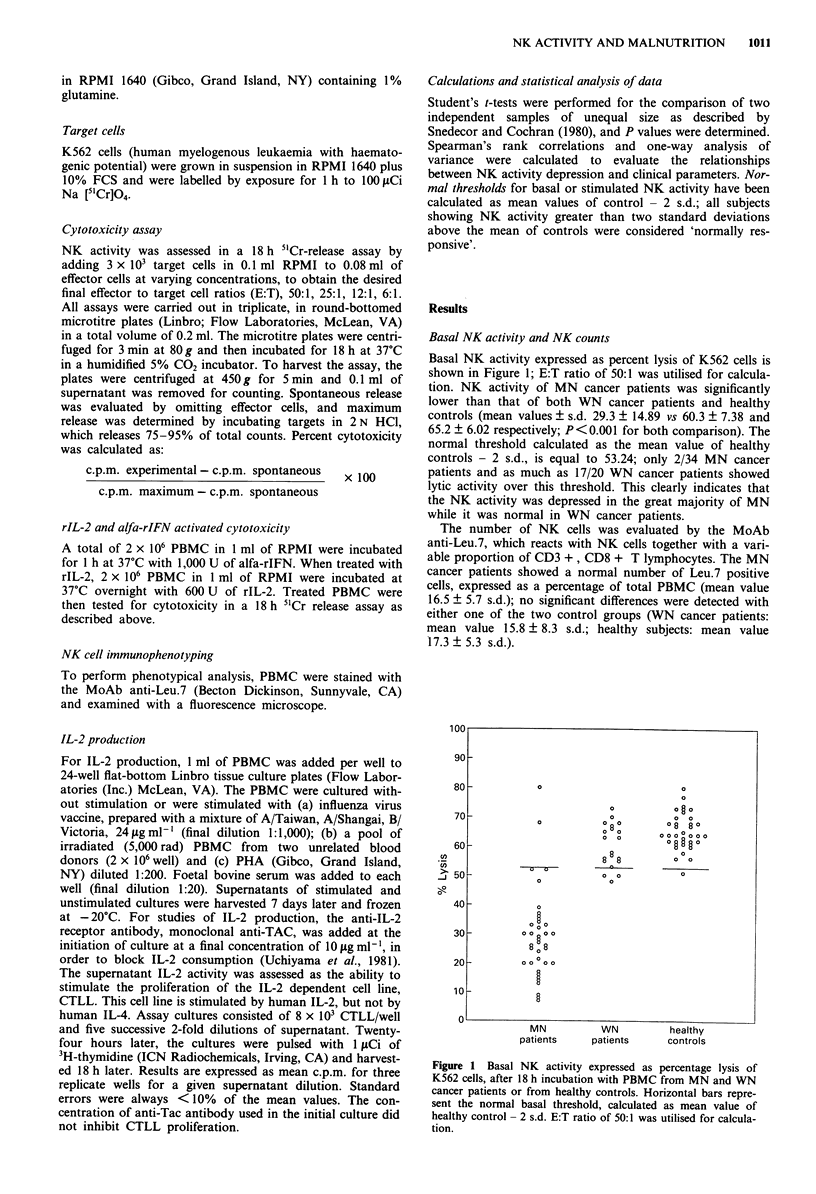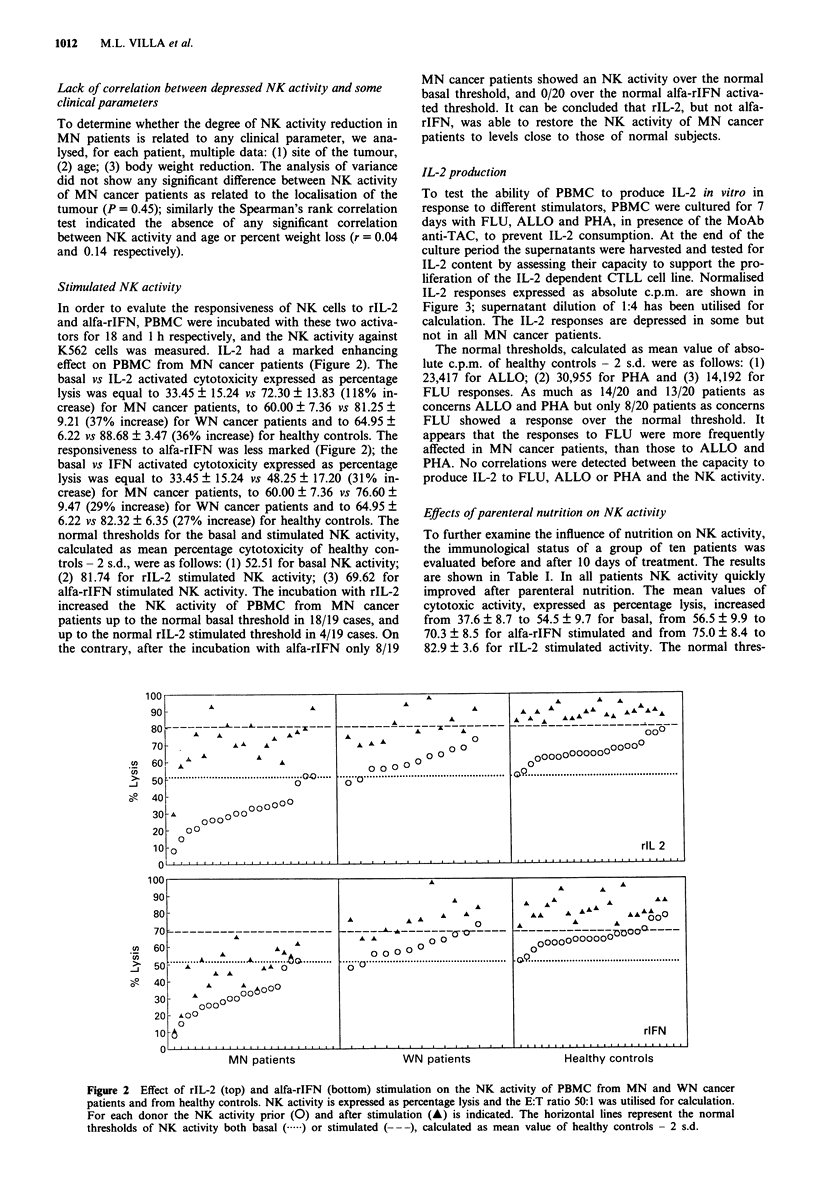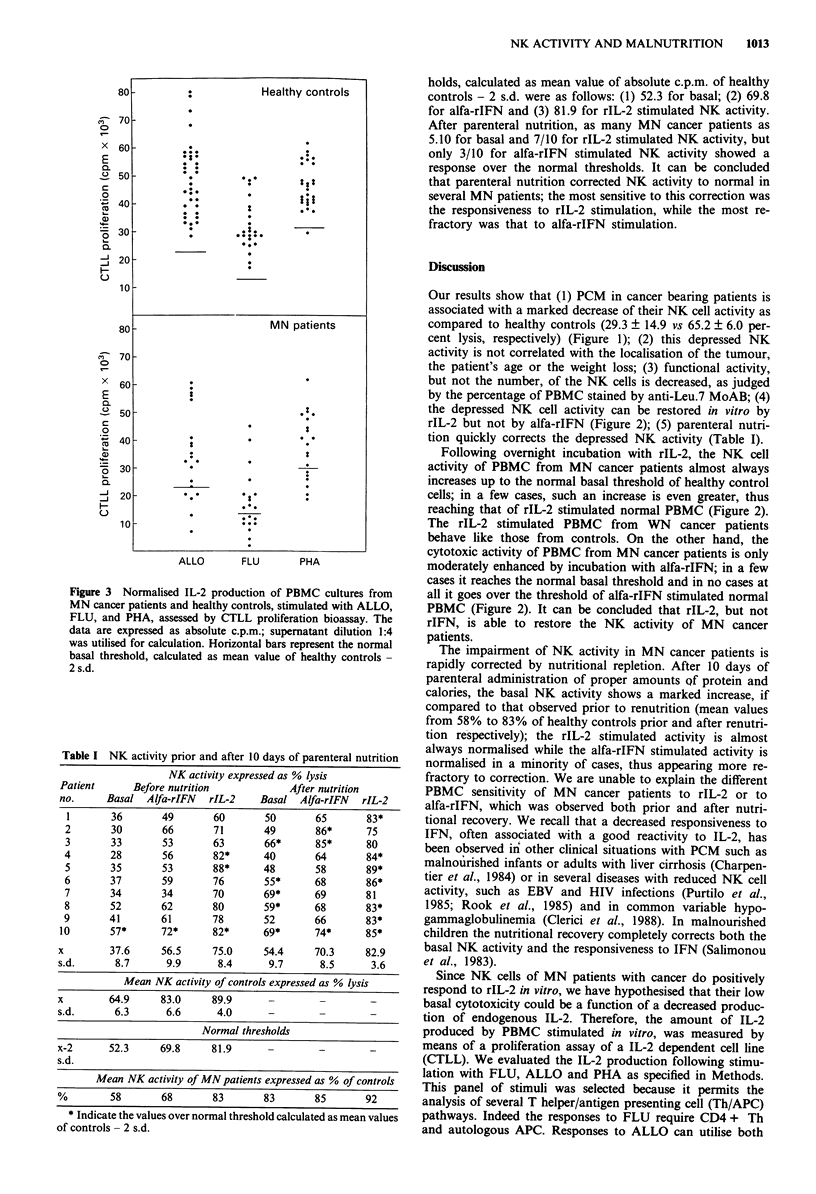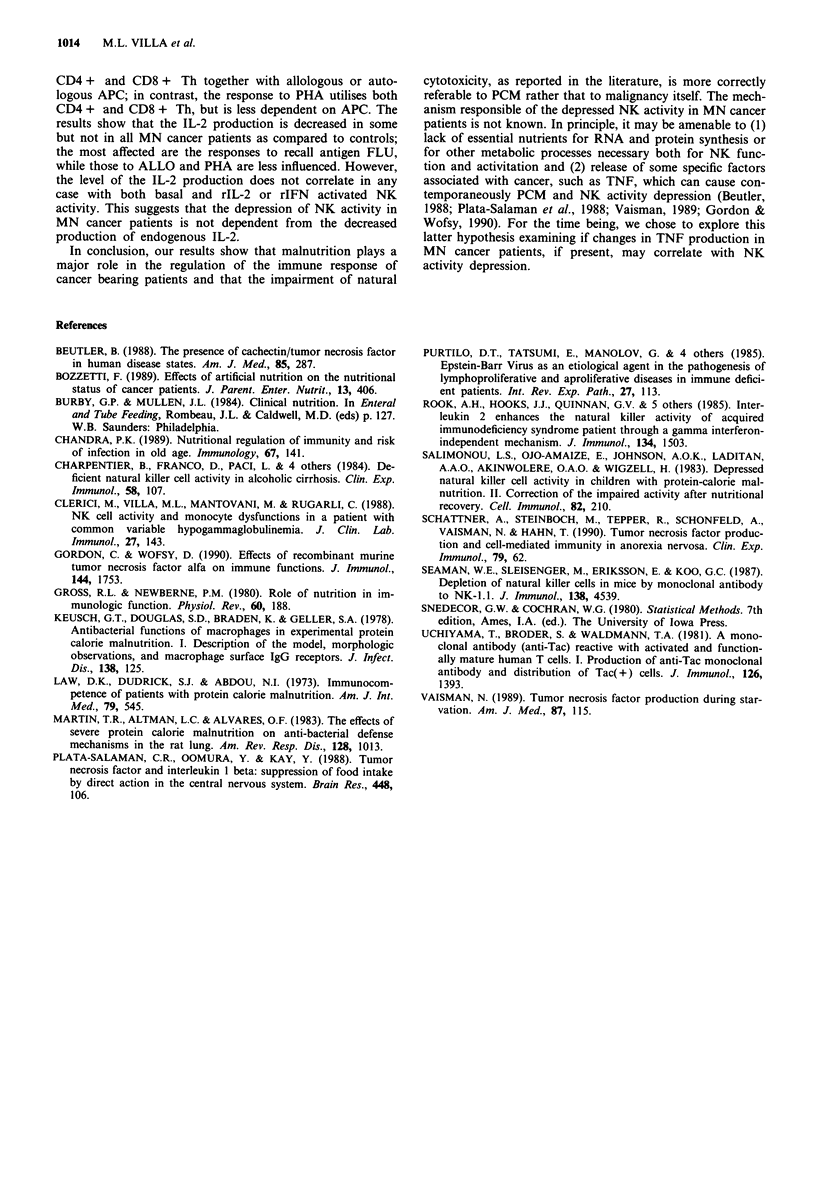# Reduced natural killer cell activity and IL-2 production in malnourished cancer patients.

**DOI:** 10.1038/bjc.1991.219

**Published:** 1991-06

**Authors:** M. L. Villa, E. Ferrario, E. Bergamasco, F. Bozzetti, L. Cozzaglio, E. Clerici

**Affiliations:** Cattedra di Immunologia, Milano, Italy.

## Abstract

Natural killer (NK) cell activity was measured in the peripheral blood mononuclear cells (PBMC) from malnourished (MN) and well-nourished (WN) cancer patients and in healthy controls. A marked depression of NK activity was observed in MN cancer patients with moderate protein-calorie malnutrition (PCM), but not in WN cancer patients nor in the healthy controls. The depression of NK activity did not correlate with the localisation of the tumour, patient's age or body weight reduction. The defective NK activity of PBMC from MN cancer patients was restored to normal by rIL-2, but not by alfa-rIFN. Parenteral nutrition of MN patients with the proper amount of proteins and calories quickly corrected the depressed NK activity, indicating a central role of malnutrition in the genesis of their immune disfunction. PBMC from MN cancer patients produced lower amounts of IL-2, as compared with healthy controls, when stimulated in vitro; the most frequently affected were the responses to recall antigens such as influenza virus vaccine (FLU), while those to allogeneic PBMC (ALLO) and phytohaemagglutinin (PHA) were less affected. However, for each patient the ability to produce IL-2 in vitro did not correlate with NK activity, thus showing how the impairment of NK activity is not subsequent to a decreased production of endogenous IL-2. In summary, it can be concluded that malnutrition, rather than malignancy, plays a major role in the immune dysfunction of cancer patients.


Br. J. Cancer (1991), 63, 1010–1014 © Macmillan Press Ltd., 1991

### Reduced natural killer cell activity and IL-2 production in malnourished cancer patients

M.L. Villa, E. Ferrario, E. Bergamasco, F. Bozzetti, L. Cozzaglio & E. Clerici

*Cattedra di Immunologia and Istituto nazionale Tumori, via Venezian 1, 20133 Milano, Italy.*

**Summary** Natural killer (NK) cell activity was measured in the peripheral blood mononuclear cells (PBMC) from malnourished (MN) and well-nourished (WN) cancer patients and in healthy controls. A marked depression of NK activity was observed in MN cancer patients with moderate protein-calorie malnutrition (PCM), but not in WN cancer patients nor in the healthy controls. The depression of NK activity did not correlate with the localisation of the tumour, patient's age or body weight reduction. The defective NK activity of PBMC from MN cancer patients was restored to normal by rIL-2, but not by alfa-rIFN. Parenteral nutrition of MN patients with the proper amount of proteins and calories quickly corrected the depressed NK activity, indicating a central role of malnutrition in the genesis of their immune disfunction. PBMC from MN cancer patients produced lower amounts of IL-2, as compared with healthy controls, when stimulated *in vitro*; the most frequently affected were the responses to recall antigens such as influenza virus vaccine (FLU), while those to allogeneic PBMC (ALLO) and phytohaemagglutinin (PHA) were less affected. However, for each patient the ability to produce IL-2 *in vitro* did not correlate with NK activity, thus showing how the impairment of NK activity is not subsequent to a decreased production of endogenous IL-2. In summary, it can be concluded that malnutrition, rather than malignancy, plays a major role in the immune dysfunction of cancer patients.

Protein calorie malnutrition (PCM), which is one of the major and most characteristic problems of cancer bearing patients, produces broad effects on the host immune defences. Defects in humoral immunity, mucosal immunity and phagocytic cell function have been identified, but the weight of evidence suggests that cell-mediated immunity is most profoundly impaired (Law *et al.*, 1973; Keusch *et al.*, 1978; Gross & Newberne, 1980; Martin *et al.*, 1983; Chandra, 1989). In particular, depressed NK activity and unresponsiveness to interferon have been reported in PBMC of infants with PCM, as well as in acute starvation (Salimonou *et al.*, 1983; Schattner *et al.*, 1990).

The aim of this research was to examine the role of PCM in the genesis of tumour associated immunodeficiency. Because of the importance of NK cells in the early defence against infectious and malignant diseases and in the *in vivo* destruction of circulating tumour emboli (Seaman *et al.*, 1987) we decided to analyse how PCM impairs NK cell cytotoxicity in cancer bearing patients. Malnourished cancer patients, showing about 10–20% reduction of body weight in the last 6 months were studied; two control groups were identified: (1) cancer patients without malnutrition and (2) normal, healthy subjects. Both the basal NK activity of PBMC and the effects of *in vitro* treatment with rIL-2 and alfa-rIFN were examined. The ability of PBMC from the same patients to produce IL-2 in response to a panel of antigens was also measured in order to recognise any possible correlation between NK and T helper (Th) cell functions.

### Materials and methods

### Special reagents

Highly purified human recombinant IL-2 (rIL-2) was provided by the Biogen (Cambridge, MA); recombinant human Interferon alfa 2a (alfa-rIFN) was provided by Roche (Basel, CH); anti-TAC and fluorescinated anti-Leu.7 monoclonal antibodies (MoAB) were from Becton Dickinson (Sunnyvale, CA).

### Patients

The patients were hospitalised at the Istituto nazionale Tumori of Milano (1987–1989) for cancer of various localisations. The patients were studied before receiving chemo- or radiotherapy. Only patients exhibiting a documented reduction of the usual body weight of 10–20% in about 6 months, were admitted to the study. None of the patients was obese before the disease. Neoplastic patients without PCM were studied and utilised as one of two control groups. A group of 37 healthy males and females, aged from 25 to 65, taken from blood donors of the Istituto nazionale Tumori, was evaluated as the second control group. All patients were thoroughly examined for anthropometric, biochemical and haematological parameters of nutritional status, which are commonly used in clinical practice (Burbry & Mullen, 1984). The characteristics of malnourished (MN) and well-nourished (WN) patients were as follows:

*WN cancer patients* 20 subjects (14 males and six females) aged from 41 to 70. No reduction of body weight during the last 6 months.

*MN cancer patients* 39 subjects (27 males and 12 females) aged from 41 to 89. Body weight reduction in the last six months between 10 and 23%. Tumour localisation was as follows: stomach, 16; liver, 6; pharynx and tongue, 5; pancreas, 3; colon-rectum, 3; others, 6.

The immune parameters of a group of ten MN cancer patients, which underwent parenteral nutrition, were also evaluated both prior and after the 10 day treatment period. The parenteral nutrition regimen included a non protein calorie load equal to 150% of the estimated resting energy expenditure with a calorie to nitrogen ratio of 150:1 and was delivered according to the commonly accepted guidelines (Bozzetti, 1989).

### Preparation of effector cells

PBMC were separated from heparinised blood samples by density centrifugation on Ficoll-Hypaque gradients (Pharmacia fine Chemicals, Uppsala, Sweden). The separated cells were washed twice in Hank's solution (Gibco, Grand Island, NJ) and the number of viable cells was determined by trypan blue exclusion and haemacytometer. Cells were resuspended

Correspondence: M.L. Villa.
Received 9 November 1990; and in revised form 11 January 1991.

in RPMI 1640 (Gibco, Grand Island, NY) containing 1% glutamine.

*Target cells*

K562 cells (human myelogenous leukaemia with haematogenic potential) were grown in suspension in RPMI 1640 plus 10% FCS and were labelled by exposure for 1 h to 100 μCi Na [$^{51}$Cr]$O_4$.

*Cytotoxicity assay*

NK activity was assessed in a 18 h $^{51}$Cr-release assay by adding $3 \times 10^3$ target cells in 0.1 ml RPMI to 0.08 ml of effector cells at varying concentrations, to obtain the desired final effector to target cell ratios (E:T), 50:1, 25:1, 12:1, 6:1. All assays were carried out in triplicate, in round-bottomed microtitre plates (Linbro; Flow Laboratories, McLean, VA) in a total volume of 0.2 ml. The microtitre plates were centrifuged for 3 min at 80 *g* and then incubated for 18 h at 37°C in a humidified 5% $CO_2$ incubator. To harvest the assay, the plates were centrifuged at 450 *g* for 5 min and 0.1 ml of supernatant was removed for counting. Spontaneous release was evaluated by omitting effector cells, and maximum release was determined by incubating targets in 2 N HCl, which releases 75–95% of total counts. Percent cytotoxicity was calculated as:

$$\frac{\text{c.p.m. experimental} - \text{c.p.m. spontaneous}}{\text{c.p.m. maximum} - \text{c.p.m. spontaneous}} \times 100$$

*rIL-2 and alfa-rIFN activated cytotoxicity*

A total of $2 \times 10^6$ PBMC in 1 ml of RPMI were incubated for 1 h at 37°C with 1,000 U of alfa-rIFN. When treated with rIL-2, $2 \times 10^6$ PBMC in 1 ml of RPMI were incubated at 37°C overnight with 600 U of rIL-2. Treated PBMC were then tested for cytotoxicity in a 18 h $^{51}$Cr release assay as described above.

*NK cell immunophenotyping*

To perform phenotypical analysis, PBMC were stained with the MoAb anti-Leu.7 (Becton Dickinson, Sunnyvale, CA) and examined with a fluorescence microscope.

*IL-2 production*

For IL-2 production, 1 ml of PBMC was added per well to 24-well flat-bottom Linbro tissue culture plates (Flow Laboratories (Inc.) McLean, VA). The PBMC were cultured without stimulation or were stimulated with (a) influenza virus vaccine, prepared with a mixture of A/Taiwan, A/Shangai, B/Victoria, 24 μg ml$^{-1}$ (final dilution 1:1,000); (b) a pool of irradiated (5,000 rad) PBMC from two unrelated blood donors ($2 \times 10^6$ well) and (c) PHA (Gibco, Grand Island, NY) diluted 1:200. Foetal bovine serum was added to each well (final dilution 1:20). Supernatants of stimulated and unstimulated cultures were harvested 7 days later and frozen at −20°C. For studies of IL-2 production, the anti-IL-2 receptor antibody, monoclonal anti-TAC, was added at the initiation of culture at a final concentration of 10 μg ml$^{-1}$, in order to block IL-2 consumption (Uchiyama *et al.*, 1981). The supernatant IL-2 activity was assessed as the ability to stimulate the proliferation of the IL-2 dependent cell line, CTLL. This cell line is stimulated by human IL-2, but not by human IL-4. Assay cultures consisted of $8 \times 10^3$ CTLL/well and five successive 2-fold dilutions of supernatant. Twenty-four hours later, the cultures were pulsed with 1 μCi of $^3$H-thymidine (ICN Radiochemicals, Irving, CA) and harvested 18 h later. Results are expressed as mean c.p.m. for three replicate wells for a given supernatant dilution. Standard errors were always <10% of the mean values. The concentration of anti-Tac antibody used in the initial culture did not inhibit CTLL proliferation.

*Calculations and statistical analysis of data*

Student's *t*-tests were performed for the comparison of two independent samples of unequal size as described by Snedecor and Cochran (1980), and *P* values were determined. Spearman's rank correlations and one-way analysis of variance were calculated to evaluate the relationships between NK activity depression and clinical parameters. *Normal thresholds* for basal or stimulated NK activity have been calculated as mean values of control − 2 s.d.; all subjects showing NK activity greater than two standard deviations above the mean of controls were considered 'normally responsive'.

### Results

*Basal NK activity and NK counts*

Basal NK activity expressed as percent lysis of K562 cells is shown in Figure 1; E:T ratio of 50:1 was utilised for calculation. NK activity of MN cancer patients was significantly lower than that of both WN cancer patients and healthy controls (mean values ± s.d. 29.3 ± 14.89 *vs* 60.3 ± 7.38 and 65.2 ± 6.02 respectively; $P < 0.001$ for both comparison). The normal threshold calculated as the mean value of healthy controls − 2 s.d., is equal to 53.24; only 2/34 MN cancer patients and as much as 17/20 WN cancer patients showed lytic activity over this threshold. This clearly indicates that the NK activity was depressed in the great majority of MN while it was normal in WN cancer patients.

The number of NK cells was evaluated by the MoAb anti-Leu.7, which reacts with NK cells together with a variable proportion of CD3 +, CD8 + T lymphocytes. The MN cancer patients showed a normal number of Leu.7 positive cells, expressed as a percentage of total PBMC (mean value 16.5 ± 5.7 s.d.); no significant differences were detected with either one of the two control groups (WN cancer patients: mean value 15.8 ± 8.3 s.d.; healthy subjects: mean value 17.3 ± 5.3 s.d.).

**Figure 1** Basal NK activity expressed as percentage lysis of K562 cells, after 18 h incubation with PBMC from MN and WN cancer patients or from healthy controls. Horizontal bars represent the normal basal threshold, calculated as mean value of healthy control − 2 s.d. E:T ratio of 50:1 was utilised for calculation.

### Lack of correlation between depressed NK activity and some clinical parameters

To determine whether the degree of NK activity reduction in MN patients is related to any clinical parameter, we analysed, for each patient, multiple data: (1) site of the tumour, (2) age; (3) body weight reduction. The analysis of variance did not show any significant difference between NK activity of MN cancer patients as related to the localisation of the tumour ($P = 0.45$); similarly the Spearman's rank correlation test indicated the absence of any significant correlation between NK activity and age or percent weight loss ($r = 0.04$ and 0.14 respectively).

### Stimulated NK activity

In order to evalute the responsiveness of NK cells to rIL-2 and alfa-rIFN, PBMC were incubated with these two activators for 18 and 1 h respectively, and the NK activity against K562 cells was measured. IL-2 had a marked enhancing effect on PBMC from MN cancer patients (Figure 2). The basal *vs* IL-2 activated cytotoxicity expressed as percentage lysis was equal to 33.45 ± 15.24 *vs* 72.30 ± 13.83 (118% increase) for MN cancer patients, to 60.00 ± 7.36 *vs* 81.25 ± 9.21 (37% increase) for WN cancer patients and to 64.95 ± 6.22 *vs* 88.68 ± 3.47 (36% increase) for healthy controls. The responsiveness to alfa-rIFN was less marked (Figure 2); the basal *vs* IFN activated cytotoxicity expressed as percentage lysis was equal to 33.45 ± 15.24 *vs* 48.25 ± 17.20 (31% increase) for MN cancer patients, to 60.00 ± 7.36 *vs* 76.60 ± 9.47 (29% increase) for WN cancer patients and to 64.95 ± 6.22 *vs* 82.32 ± 6.35 (27% increase) for healthy controls. The normal thresholds for the basal and stimulated NK activity, calculated as mean percentage cytotoxicity of healthy controls – 2 s.d., were as follows: (1) 52.51 for basal NK activity; (2) 81.74 for rIL-2 stimulated NK activity; (3) 69.62 for alfa-rIFN stimulated NK activity. The incubation with rIL-2 increased the NK activity of PBMC from MN cancer patients up to the normal basal threshold in 18/19 cases, and up to the normal rIL-2 stimulated threshold in 4/19 cases. On the contrary, after the incubation with alfa-rIFN only 8/19 MN cancer patients showed an NK activity over the normal basal threshold, and 0/20 over the normal alfa-rIFN activated threshold. It can be concluded that rIL-2, but not alfa-rIFN, was able to restore the NK activity of MN cancer patients to levels close to those of normal subjects.

### IL-2 production

To test the ability of PBMC to produce IL-2 *in vitro* in response to different stimulators, PBMC were cultured for 7 days with FLU, ALLO and PHA, in presence of the MoAb anti-TAC, to prevent IL-2 consumption. At the end of the culture period the supernatants were harvested and tested for IL-2 content by assessing their capacity to support the proliferation of the IL-2 dependent CTLL cell line. Normalised IL-2 responses expressed as absolute c.p.m. are shown in Figure 3; supernatant dilution of 1:4 has been utilised for calculation. The IL-2 responses are depressed in some but not in all MN cancer patients.

The normal thresholds, calculated as mean value of absolute c.p.m. of healthy controls – 2 s.d. were as follows: (1) 23,417 for ALLO; (2) 30,955 for PHA and (3) 14,192 for FLU responses. As much as 14/20 and 13/20 patients as concerns ALLO and PHA but only 8/20 patients as concerns FLU showed a response over the normal threshold. It appears that the responses to FLU were more frequently affected in MN cancer patients, than those to ALLO and PHA. No correlations were detected between the capacity to produce IL-2 to FLU, ALLO or PHA and the NK activity.

### Effects of parenteral nutrition on NK activity

To further examine the influence of nutrition on NK activity, the immunological status of a group of ten patients was evaluated before and after 10 days of treatment. The results are shown in Table I. In all patients NK activity quickly improved after parenteral nutrition. The mean values of cytotoxic activity, expressed as percentage lysis, increased from 37.6 ± 8.7 to 54.5 ± 9.7 for basal, from 56.5 ± 9.9 to 70.3 ± 8.5 for alfa-rIFN stimulated and from 75.0 ± 8.4 to 82.9 ± 3.6 for rIL-2 stimulated activity. The normal thres-

**Figure 2** Effect of rIL-2 (top) and alfa-rIFN (bottom) stimulation on the NK activity of PBMC from MN and WN cancer patients and from healthy controls. NK activity is expressed as percentage lysis and the E:T ratio 50:1 was utilised for calculation. For each donor the NK activity prior (O) and after stimulation (▲) is indicated. The horizontal lines represent the normal thresholds of NK activity both basal (·····) or stimulated (– – –), calculated as mean value of healthy controls – 2 s.d.

**Figure 3** Normalised IL-2 production of PBMC cultures from MN cancer patients and healthy controls, stimulated with ALLO, FLU, and PHA, assessed by CTLL proliferation bioassay. The data are expressed as absolute c.p.m.; supernatant dilution 1:4 was utilised for calculation. Horizontal bars represent the normal basal threshold, calculated as mean value of healthy controls − 2 s.d.

**Table I** NK activity prior and after 10 days of parenteral nutrition

| Patient no. | *NK activity expressed as % lysis* Before nutrition Basal | Alfa-rIFN | rIL-2 | After nutrition Basal | Alfa-rIFN | rIL-2 |
|---|---|---|---|---|---|---|
| 1 | 36 | 49 | 60 | 50 | 65 | 83* |
| 2 | 30 | 66 | 71 | 49 | 86* | 75 |
| 3 | 33 | 53 | 63 | 66* | 85* | 80 |
| 4 | 28 | 56 | 82* | 40 | 64 | 84* |
| 5 | 35 | 53 | 88* | 48 | 58 | 89* |
| 6 | 37 | 59 | 76 | 55* | 68 | 86* |
| 7 | 34 | 34 | 70 | 69* | 69 | 81 |
| 8 | 52 | 62 | 80 | 59* | 68 | 83* |
| 9 | 41 | 61 | 78 | 52 | 66 | 83* |
| 10 | 57* | 72* | 82* | 69* | 74* | 85* |
| x | 37.6 | 56.5 | 75.0 | 54.4 | 70.3 | 82.9 |
| s.d. | 8.7 | 9.9 | 8.4 | 9.7 | 8.5 | 3.6 |
| | *Mean NK activity of controls expressed as % lysis* | | | | | |
| x | 64.9 | 83.0 | 89.9 | – | – | – |
| s.d. | 6.3 | 6.6 | 4.0 | – | – | – |
| | *Normal thresholds* | | | | | |
| x-2 s.d. | 52.3 | 69.8 | 81.9 | – | – | – |
| | *Mean NK activity of MN patients expressed as % of controls* | | | | | |
| % | 58 | 68 | 83 | 83 | 85 | 92 |

* Indicate the values over normal threshold calculated as mean values of controls − 2 s.d.

holds, calculated as mean value of absolute c.p.m. of healthy controls − 2 s.d. were as follows: (1) 52.3 for basal; (2) 69.8 for alfa-rIFN and (3) 81.9 for rIL-2 stimulated NK activity. After parenteral nutrition, as many MN cancer patients as 5.10 for basal and 7/10 for rIL-2 stimulated NK activity, but only 3/10 for alfa-rIFN stimulated NK activity showed a response over the normal thresholds. It can be concluded that parenteral nutrition corrected NK activity to normal in several MN patients; the most sensitive to this correction was the responsiveness to rIL-2 stimulation, while the most refractory was that to alfa-rIFN stimulation.

### Discussion

Our results show that (1) PCM in cancer bearing patients is associated with a marked decrease of their NK cell activity as compared to healthy controls (29.3 ± 14.9 *vs* 65.2 ± 6.0 percent lysis, respectively) (Figure 1); (2) this depressed NK activity is not correlated with the localisation of the tumour, the patient's age or the weight loss; (3) functional activity, but not the number, of the NK cells is decreased, as judged by the percentage of PBMC stained by anti-Leu.7 MoAB; (4) the depressed NK cell activity can be restored *in vitro* by rIL-2 but not by alfa-rIFN (Figure 2); (5) parenteral nutrition quickly corrects the depressed NK activity (Table I).

Following overnight incubation with rIL-2, the NK cell activity of PBMC from MN cancer patients almost always increases up to the normal basal threshold of healthy control cells; in a few cases, such an increase is even greater, thus reaching that of rIL-2 stimulated normal PBMC (Figure 2). The rIL-2 stimulated PBMC from WN cancer patients behave like those from controls. On the other hand, the cytotoxic activity of PBMC from MN cancer patients is only moderately enhanced by incubation with alfa-rIFN; in a few cases it reaches the normal basal threshold and in no cases at all it goes over the threshold of alfa-rIFN stimulated normal PBMC (Figure 2). It can be concluded that rIL-2, but not rIFN, is able to restore the NK activity of MN cancer patients.

The impairment of NK activity in MN cancer patients is rapidly corrected by nutritional repletion. After 10 days of parenteral administration of proper amounts of protein and calories, the basal NK activity shows a marked increase, if compared to that observed prior to renutrition (mean values from 58% to 83% of healthy controls prior and after renutrition respectively); the rIL-2 stimulated activity is almost always normalised while the alfa-rIFN stimulated activity is normalised in a minority of cases, thus appearing more refractory to correction. We are unable to explain the different PBMC sensitivity of MN cancer patients to rIL-2 or to alfa-rIFN, which was observed both prior and after nutritional recovery. We recall that a decreased responsiveness to IFN, often associated with a good reactivity to IL-2, has been observed in other clinical situations with PCM such as malnourished infants or adults with liver cirrhosis (Charpentier *et al.*, 1984) or in several diseases with reduced NK cell activity, such as EBV and HIV infections (Purtilo *et al.*, 1985; Rook *et al.*, 1985) and in common variable hypogammaglobulinemia (Clerici *et al.*, 1988). In malnourished children the nutritional recovery completely corrects both the basal NK activity and the responsiveness to IFN (Salimonou *et al.*, 1983).

Since NK cells of MN patients with cancer do positively respond to rIL-2 *in vitro*, we have hypothesised that their low basal cytotoxicity could be a function of a decreased production of endogenous IL-2. Therefore, the amount of IL-2 produced by PBMC stimulated *in vitro*, was measured by means of a proliferation assay of a IL-2 dependent cell line (CTLL). We evaluated the IL-2 production following stimulation with FLU, ALLO and PHA as specified in Methods. This panel of stimuli was selected because it permits the analysis of several T helper/antigen presenting cell (Th/APC) pathways. Indeed the responses to FLU require CD4 + Th and autologous APC. Responses to ALLO can utilise both

CD4+ and CD8+ Th together with allologous or autologous APC; in contrast, the response to PHA utilises both CD4+ and CD8+ Th, but is less dependent on APC. The results show that the IL-2 production is decreased in some but not in all MN cancer patients as compared to controls; the most affected are the responses to recall antigen FLU, while those to ALLO and PHA are less influenced. However, the level of the IL-2 production does not correlate in any case with both basal and rIL-2 or rIFN activated NK activity. This suggests that the depression of NK activity in MN cancer patients is not dependent from the decreased production of endogenous IL-2.

In conclusion, our results show that malnutrition plays a major role in the regulation of the immune response of cancer bearing patients and that the impairment of natural cytotoxicity, as reported in the literature, is more correctly referable to PCM rather that to malignancy itself. The mechanism responsible of the depressed NK activity in MN cancer patients is not known. In principle, it may be amenable to (1) lack of essential nutrients for RNA and protein synthesis or for other metabolic processes necessary both for NK function and activitation and (2) release of some specific factors associated with cancer, such as TNF, which can cause contemporaneously PCM and NK activity depression (Beutler, 1988; Plata-Salaman *et al.*, 1988; Vaisman, 1989; Gordon & Wofsy, 1990). For the time being, we chose to explore this latter hypothesis examining if changes in TNF production in MN cancer patients, if present, may correlate with NK activity depression.

### References

BEUTLER, B. (1988). The presence of cachectin/tumor necrosis factor in human disease states. *Am. J. Med.*, **85**, 287.

BOZZETTI, F. (1989). Effects of artificial nutrition on the nutritional status of cancer patients. *J. Parent. Enter. Nutrit.*, **13**, 406.

BURBY, G.P. & MULLEN, J.L. (1984). Clinical nutrition. In *Enteral and Tube Feeding*, Rombeau, J.L. & Caldwell, M.D. (eds) p. 127. W.B. Saunders: Philadelphia.

CHANDRA, P.K. (1989). Nutritional regulation of immunity and risk of infection in old age. *Immunology*, **67**, 141.

CHARPENTIER, B., FRANCO, D., PACI, L. & 4 others (1984). Deficient natural killer cell activity in alcoholic cirrhosis. *Clin. Exp. Immunol.*, **58**, 107.

CLERICI, M., VILLA, M.L., MANTOVANI, M. & RUGARLI, C. (1988). NK cell activity and monocyte dysfunctions in a patient with common variable hypogammaglobulinemia. *J. Clin. Lab. Immunol.*, **27**, 143.

GORDON, C. & WOFSY, D. (1990). Effects of recombinant murine tumor necrosis factor alfa on immune functions. *J. Immunol.*, **144**, 1753.

GROSS, R.L. & NEWBERNE, P.M. (1980). Role of nutrition in immunologic function. *Physiol. Rev.*, **60**, 188.

KEUSCH, G.T., DOUGLAS, S.D., BRADEN, K. & GELLER, S.A. (1978). Antibacterial functions of macrophages in experimental protein calorie malnutrition. I. Description of the model, morphologic observations, and macrophage surface IgG receptors. *J. Infect. Dis.*, **138**, 125.

LAW, D.K., DUDRICK, S.J. & ABDOU, N.I. (1973). Immunocompetence of patients with protein calorie malnutrition. *Am. J. Int. Med.*, **79**, 545.

MARTIN, T.R., ALTMAN, L.C. & ALVARES, O.F. (1983). The effects of severe protein calorie malnutrition on anti-bacterial defense mechanisms in the rat lung. *Am. Rev. Resp. Dis.*, **128**, 1013.

PLATA-SALAMAN, C.R., OOMURA, Y. & KAY, Y. (1988). Tumor necrosis factor and interleukin 1 beta: suppression of food intake by direct action in the central nervous system. *Brain Res.*, **448**, 106.

PURTILO, D.T., TATSUMI, E., MANOLOV, G. & 4 others (1985). Epstein-Barr Virus as an etiological agent in the pathogenesis of lymphoproliferative and aproliferative diseases in immune deficient patients. *Int. Rev. Exp. Path.*, **27**, 113.

ROOK, A.H., HOOKS, J.J., QUINNAN, G.V. & 5 others (1985). Interleukin 2 enhances the natural killer activity of acquired immunodeficiency syndrome patient through a gamma interferon-independent mechanism. *J. Immunol.*, **134**, 1503.

SALIMONOU, L.S., OJO-AMAIZE, E., JOHNSON, A.O.K., LADITAN, A.A.O., AKINWOLERE, O.A.O. & WIGZELL, H. (1983). Depressed natural killer cell activity in children with protein-calorie malnutrition. II. Correction of the impaired activity after nutritional recovery. *Cell. Immunol.*, **82**, 210.

SCHATTNER, A., STEINBOCH, M., TEPPER, R., SCHONFELD, A., VAISMAN, N. & HAHN, T. (1990). Tumor necrosis factor production and cell-mediated immunity in anorexia nervosa. *Clin. Exp. Immunol.*, **79**, 62.

SEAMAN, W.E., SLEISENGER, M., ERIKSSON, E. & KOO, G.C. (1987). Depletion of natural killer cells in mice by monoclonal antibody to NK-1.1. *J. Immunol.*, **138**, 4539.

SNEDECOR, G.W. & COCHRAN, W.G. (1980). *Statistical Methods*. 7th edition, Ames, I.A. (ed.). The University of Iowa Press.

UCHIYAMA, T., BRODER, S. & WALDMANN, T.A. (1981). A monoclonal antibody (anti-Tac) reactive with activated and functionally mature human T cells. I. Production of anti-Tac monoclonal antibody and distribution of Tac(+) cells. *J. Immunol.*, **126**, 1393.

VAISMAN, N. (1989). Tumor necrosis factor production during starvation. *Am. J. Med.*, **87**, 115.